# A pilot surveillance report of SARS-CoV-2 rapid antigen test results among volunteers in Germany, 1st week of July 2022

**DOI:** 10.1007/s15010-022-01931-7

**Published:** 2022-10-24

**Authors:** Jannik Stemler, Jon Salmanton-García, Ben Weise, Christina Többen, Carolin Joisten, Julian Fleig, Oliver A. Cornely, on behalf of the VACCELERATE Consortium

**Affiliations:** 1 Faculty of Medicine and University Hospital Cologne, Translational Research, Cologne Excellence Cluster on Cellular Stress Responses in Aging-Associated Diseases (CECAD), University of Cologne, Herderstr. 52-54, 50931 Cologne, Germany; 2 Faculty of Medicine and University Hospital Cologne, Department I of Internal Medicine, Center for Integrated Oncology Aachen Bonn Cologne Duesseldorf (CIO ABCD) and Excellence Center for Medical Mycology (ECMM), University of Cologne, Cologne, Germany; 3 German Centre for Infection Research (DZIF), Partner Site Bonn-Cologne, Cologne, Germany; 4 Faculty of Medicine and University Hospital Cologne, Clinical Trials Centre Cologne (ZKS Köln), University of Cologne, Cologne, Germany

**Keywords:** SARS-CoV-2, Point prevalence, Surveillance, Pandemic preparedness, Self-testing, Rapid antigen test

## Abstract

**Purpose:**

We hypothesized that SARS-CoV-2 infection numbers reported by governmental institutions are underestimated due to high dark figures as only results from polymerase chain reaction (PCR) tests are incorporated in governmental statistics and testing capacities were further restricted as of July, 2022.

**Methods:**

A point prevalence investigation was piloted by rapid antigen testing (RAT) among participants of the VACCELERATE volunteer registry. 2400 volunteers were contacted, of which 500 received a RAT including instructions for self-testing in the first week of July, 2022. Results were self-reported via e-mail.

**Results:**

419 valid RAT results were collected until July 7th, 2022. Between July-1 and July-7, 2022, 7/419 (1.67%) tests were positive. Compared to reports of the German Federal Government, our results suggest a more than twofold higher prevalence. Three out of seven positive individuals did not have a PCR test and are therefore likely not to be displayed in governmental statistics.

**Conclusion:**

Our findings imply that the actual prevalence of SARS-CoV-2 may be higher than detected by current surveillance systems, so that current pandemic surveillance and testing strategies may be adapted.

## Introduction

With the global surge of Severe Acute Respiratory Syndrome-Coronavirus-2 (SARS-CoV-2) *Omicron* subvariants BA.4 and BA.5, its enhanced antibody escape, and reduced infection control and prevention measures in most countries, infections due to SARS-CoV-2 rose also among vaccinated and recovered individuals [1, 2]. At the same time, hospitalizations and death rates did not seem to rise significantly, meaning that the infections do not affect most people severely, but still influence people’s daily lives and cause massive work absences [3].

In Germany, the reported incidence of laboratory-based diagnosis, i.e., primarily via polymerase chain reaction (PCR) of SARS-CoV-2 infections increased rapidly since the beginning of June, 2022 [4]. Epidemiological predominance of the *Omicron* BA.5 subvariant was noted from the end of the same month, while the variant-of-concern (VOC) *Omicron* has been circulating with a share of 99% among the general population already for more than five months [5]. The numbers reported by the Federal Government are likely to differ substantially from the real incidence as most SARS-CoV-2-infected individuals are not tested via PCR anymore according to the national testing strategy by the Federal Ministry of Health (MOH) [6]. They were rather diagnosed by rapid antigen test (RAT), which may not be added to the national statistics.

We aimed to describe the point prevalence of SARS-CoV-2 infections among a representative random sample of volunteers in Germany, to elucidate a potential underreporting in notified numbers of the RKI or MOH, and to estimate the actual prevalence of SARS-CoV-2 infections.

## Methods and results

The VACCELERATE Volunteer Registry is an online registry under the umbrella of the VACCELERATE consortium, where people interested in participating in SARS-CoV-2 clinical studies can sign up via an online survey (www.vaccelerate.eu/volunteer-registry) [7]. The VACCELERATE Volunteer Registry was approved by the Ethics Committee of the Medical Faculty of the University of Cologne (Cologne, Germany) (Study number 20–1536) and currently is active in 15 European countries.

**Fig. 1:**
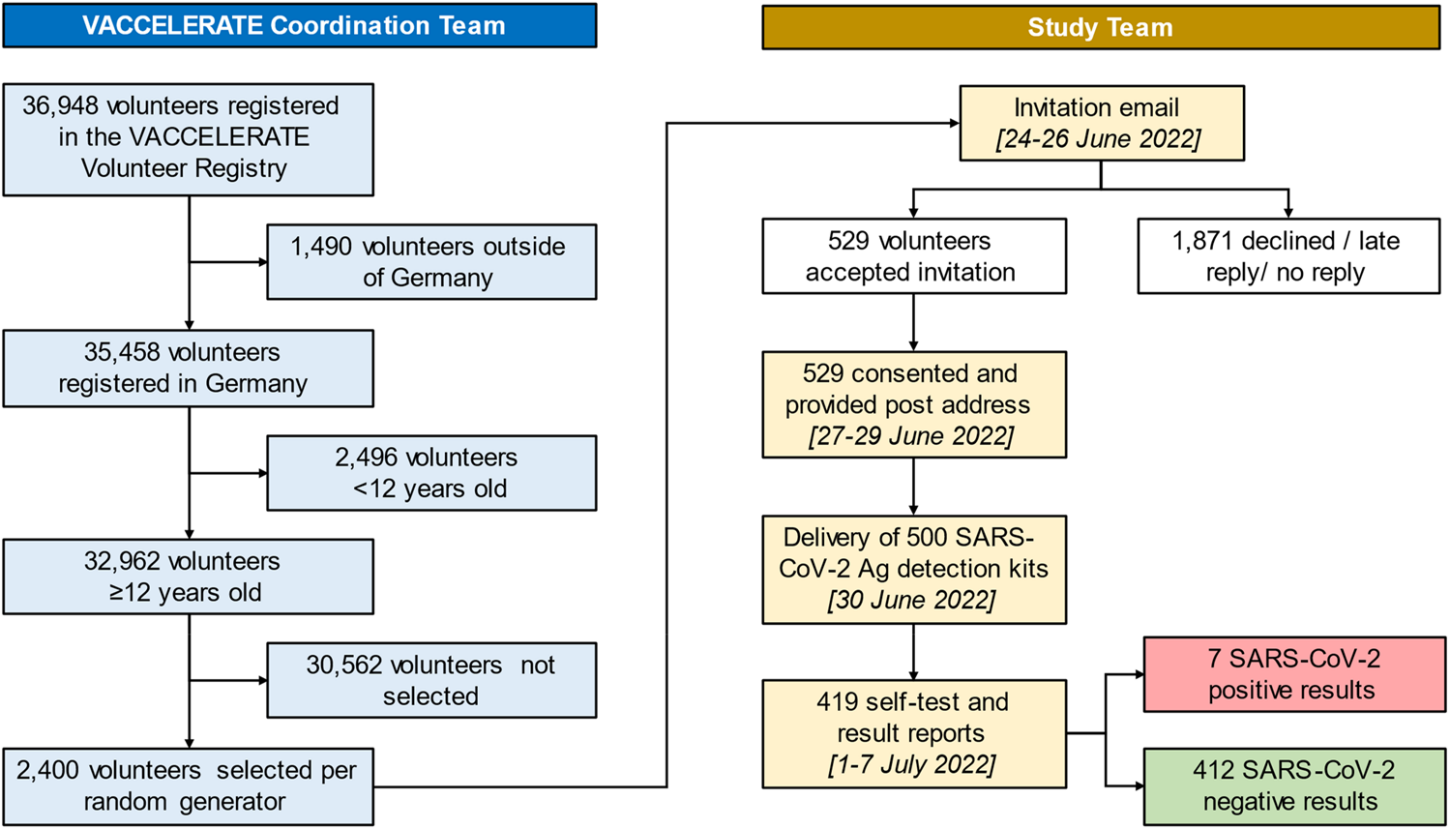
Methodology and enrollment flow chart

Among those registered in Germany, 32,962 volunteers ≥ 12 years were randomly selected with same odds for each registered participant to be shortlisted and selected for this evaluation (0.073%). Selected volunteers were invited to participate in this survey. A sample of 2400 registered individuals ≥ 12 years of age was contacted via email between June 23rd and 25th, 2022 (Fig. 1).

If a volunteer desired to participate, postal address was collected to allow sending of a RAT kit. Then, a SARS-CoV-2 rapid test (“NEWGENE COVID-19 Antigen Test Kit Schnelltest”, COVID-19-NG21, New Gene Bioengineering Co. Ldt., Hangzhou, People’s Republic of China; sensitivity of 100% with Ct ≤ 25, manufacturer specificity 99.2% [8]) was sent with instructions on how to self-test properly on the day of delivery. Five-hundred test-kits were sent on June 30th, 2022.

Results were then self-reported via email and collected until July 8th, 2022 and, therefore, resemble a point prevalence of July 1st–July 7th, 2022. Overall, 57% of participants were female, median age of participants was 44 years (Table 1). Up to this point, 419 participants had submitted a result, among those, seven participants tested positive for SARS-COV-2 via RAT (7/419, 1.67%). Six RAT test kits were damaged and, thus, unevaluable or were non-readable.

**Table 1:** Demographic data and test results of participants

	Overall		RAT Positive		RAT Negative	
	*n*	%	*n*	%	*n*	%
Age, median (IQR) [range]	44 (34–57) [12–80]		43 (28–61) [12–65]		44 (34–57) [12–80]	
12–17	11	2·6	1	14·3	10	2·4
18–30	77	18·4	1	14·3	76	18·4
31–40	89	21·2	1	14·3	88	21·4
41–50	74	17·7	1	14·3	73	17·7
51–60	102	24·3	1	14·3	101	24·5
61–70	54	12·9	2	28·6	52	12·6
71–80	12	2·9	0	0·0	12	2·9
Gender						
Female	239	57	6	85·7	233	56·6
Male	180	43	1	14·3	179	43·4
SARS-CoV-2 rapid antigen test results						
Positive	7	1·7	7	100·0	–	–
Already known to participant	7	1·7	7	100·0	–	–
Reported to government—yes	4	1·0	4	57·1	–	–
Reported to government—no	3	0·7	3	42·8		
Negative	412	98·3	–	–	412	100·0

**Fig. 2:**
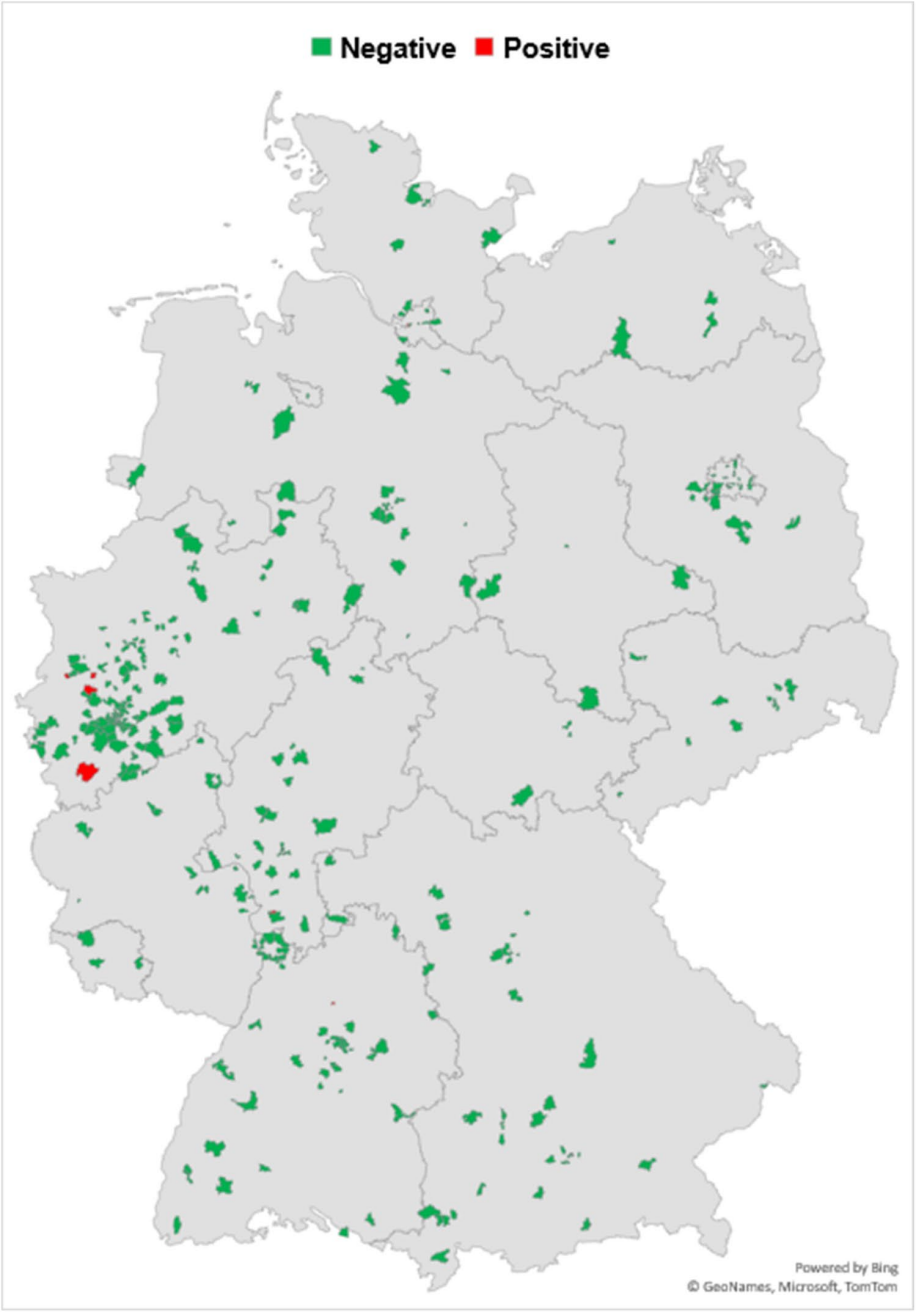
Geographical distribution of test results in Germany, July 1st–4th, 2022. Explanation: the high number of participants including four individuals tested positive in the western part of Germany may explained by (1) a higher population density in the Rhein-Ruhr area and (2) by the site of the VACCELERATE Volunteer registry coordination office in Cologne, Germany and, therefore, a locally higher number of registered participants. The comparably high number of participants tested positive (four) in this area may be incidental and cannot be extrapolated from general incidence in Western Germany (state of North Rhine-Westphalia) at the time of this study

Among the SARS-CoV-2-positive individuals, information was collected on whether infection was already known and whether a PCR result had been performed to calculate any discrepancy between our survey and the governmental reports. All 7 RAT positive individuals had known about their infection before, 4/7 (57.1%) also had a registered positive PCR result, meaning that 42.8% of participants with a positive RAT were not represented in the federal statistics. Among RAT positive individuals, all 7 had received at least three doses of SARS-CoV-2 vaccine. None of them was hospitalized with COVID-19. Geographic distribution of participants was captured via postal code and showed an even distribution throughout Germany (Fig. 2). Evaluation of regional 7-day incidence compared to the weekly governmental report was not meaningful due to small sample number for each region [9].

We report a point prevalence rate for SARS-CoV-2 infection of 1.67% (7/419, equals 1670 per 100,000 inhabitants) in our dataset. If calculated as a value for a representative random sample of individuals living in Germany, it would describe a 7-day-case number of 1,390,059 (calculated with the inhabitant number of 83,237,124 as of 31-Dec-2021 according to the Federal Statistical Office (Statistisches Bundesamt)). This means a 2.39 times higher number than the reports of the RKI dashboard (https://corona.rki.de/) by July 9th, 2022 (7-day incidence of 700.3 and 7-day case number 582,315).

## Discussion and implications

With increasing SARS-CoV-2 infection numbers among immunized individuals due to the *Omicron* variant, the concurrent antibody escape of the BA.5 subvariant and without any contact precautions in place, SARS-CoV-2 infections spread even more rapidly than previously observed. This affects society with extremely high absolute numbers of simultaneously infected individuals. This can be observed as the current pandemic situation in Germany has affected the so-called critical infrastructure (healthcare, transport sector, industry, agriculture) which is burdened by massive personnel sick leave numbers. Recently, Germany faced noticeable delay of goods and service delivery. At the same time, numbers of hospital admissions and COVID-19 patients in need of intensive care were increasing as of July 8th, 2022 in Germany, aggravating the burden on the health care system [10]. Many infected individuals have been re-infected with the *Omicron* VOC despite previous SARS-CoV-2 vaccination [11]. Duration of antibody-mediated immunity seems to persist only shortly for SARS-CoV-2, while studies suggest that previous vaccination and infection—a so-called hybrid immunity—seems to protect best from re-infection [12, 13].

Facing this situation, political action regarding infection control prevention measures seems indispensable. With the third change to the National Testing Strategy of the German Federal Ministry of Health, capacities for SARS-CoV-2 testing were aimed to be utilized more precisely. Therefore, RAT will only be free-of-charge for defined risk groups from July 1st, 2022 in Germany [14]. This may further decrease the reliability of the reported SARS-CoV-2 incidence. Otherwise, the possibility to register self-tested RAT results in the governmental database would allow more precise reporting. Recent regional sewage water sampling of SARS-CoV-2 has suggested a twofold higher rate of SARS-CoV-2 infections than the reported incidence [15], which is in line with our finding of a more than twofold higher prevalence.

Policy makers may use pilot projects like ours to adjust strategies, as well as to guide the local or timely implementation of infection prevention measures. In an expansion of our project, larger and thus more representative groups may be tested regularly in the future in point prevalence studies, while at the same time systematic surveillance of sewage water may be helpful to complement real-time pandemic monitoring [16].

Our pilot investigation has some limitations. First, we assessed a point prevalence with a rather low participant number. Second, our investigation excluded children below 12 years of age, who may show different epidemiological patterns. Third, self-testing bears a potential for inferior test performance [17]. Fourth, the geographic distribution of reported test results varies substantially and regional epidemiological patterns regarding prevalence of SARS-CoV-2 infections are not displayed.

To conclude, determination of exact numbers of incidence and prevalence in a rapidly globally spreading disease is almost impossible [18]. Overall, we show that results of random point prevalence studies differ largely from reported governmental data. Such evaluations as well as wastewater analyses may help to determine more precisely a status of the pandemic, when mass testing is not feasible anymore due to capacities or the economic and logistical burden of such testing strategies, or when people infected with SARS-CoV-2 may refrain from confirmatory testing.
